# Clinical, Laboratory and Radiographic Features of Patients with Pneumonia and Parapneumonic Effusions

**DOI:** 10.3889/oamjms.2016.091

**Published:** 2016-08-23

**Authors:** Sanja Petrusevska-Marinkovic, Irena Kondova-Topuzovska, Zvonko Milenkovic, Goran Kondov, Ankica Anastasovska

**Affiliations:** 1 *University Infectious Diseases Clinic, Medical Faculty, Ss Cyril and Methodius University of Skopje, Skopje, Republic of Macedonia*; 2 *University Thoracocardiovascular Surgery Clinic, Medical Faculty, Ss Cyril and Methodius University of Skopje, Skopje, Republic of Macedonia*

**Keywords:** community-acquired pneumonia (CAP), parapneumonic effusion, empyema, clinical features, laboratory features, radiographic features

## Abstract

**BACKGROUND:**

Parapneumonic effusions complicating pneumonia are associated with increased morbidity and mortality.

**AIM:**

To determine the role of the clinical, laboratory and radiographic features to the differential diagnosis of patients with community- acquired pneumonia (CAP) without effusion, uncomplicated parapneumonic effusion (UCPPE) and complicated parapneumonic effusion (CPPE).

**MATERIAL AND METHODS:**

We analysed 148 patients with CAP without effusion, 50 with UCPPE and 44 with CPPE. In three groups of patients, the majority was male patients (58.11%, 58%, 61.36%) consequently.

**RESULTS:**

The chronic heart failure was the most common comorbidity in a group with CAP (28; 18.92%) and UCPPE (7; 14%), alcoholism (12;12.77%) in a group with CPPE. Patients with CPPE had significantly longer fever compared to patients with CAP without effusion (p = 0.003). Pleuritic chest pain (86.36%) and dyspnea (88.64%) were the most common symptoms in CPPE, then to group with UCPPE (60%; 52%), and in CAP without effusion (25.68%; 47,97%). Diffuse pulmonary changes were detected more frequently in the group with CAP without effusion compared with the group with CPPE (64.86 % vs. 27.27 %), while the segment lung changes were more common in patients with CPPE (50% vs. 20.27%). Patients with CPPE were significant with higher erythrocytes sedimentation rate (ESR), white blood cells (WBC) and serum C- reactive protein (CRP) than it the other two groups (p = 0.00090, p = 0.01, p= 0.000065).

**CONCLUSION:**

Proper analysis of clinical, laboratory and radiographic features of patients with CAP and parapneumonic effusion can prevent mismanagement in these patients and will reduce morbidity and mortality.

## Introduction

Pleural effusion represents a common complication of community- acquired pneumonia. Parapneumonic effusions occur in 20- 40% of patients who are hospitalised with pneumonia [1]. The mortality rate in patients with parapneumonic effusions is higher than that in patients with pneumonia without parapneumonic effusion [1]. Some of the excess mortality is due to the mismanagement of pneumonia and parapneumonic effusion [1, 2]. The clinical classification of parapneumonic effusions identifies three groups with a distinct prognosis [1-3]. First, there is the simple or uncomplicated parapneumonic effusion (UCPPE), which should resolve with appropriate antibiotic therapy without any consequences within the pleural space. Second, there is a complicated parapneumonic effusion (CPPE) denoting the presence of bacterial infection in the pleural space with an associated inflammatory response. A complicated effusion sometimes requires pleural drainage to resolve and without treatment may progress to an empyema [1-4]. CPPE occurs in 10% of all patients hospitalised with effusion [4].

Finally, empyema represents frank pus in the pleural space requires pleural drainage and may also require surgical treatment [3-5]. About 60% of empyemas are related to a primary pneumonic process; therefore risk factors for pleural infection are similar to those for pneumonia [1, 6]. Independent risk factors for the development of empyema include diabetes, alcohol and intravenous abuse, immunosuppression, gastro- oesophagal reflux disease, aspiration and poor oral hygiene [4, 7, 8]. After a variable time interval, pleural infection enters an organizing, stage characterised by fibroblast proliferation and the development of solid fibrous peel. This inhibits lung reexpansion and usually necessitates surgical thoracotomy and decortication [7, 8].

There is considerable variation in the course and aggressiveness of parapneumonic effusions; therefore an understanding of its progression is important [4]. Along out with increased mortality, complicated parapneumonic effusion and empyema often necessitate prolonged treatment, longer hospital stay and interventions. Thus, identification of these patients and prompt management is critical.

Recent studies have shown that relying on clinical findings alone do not predict the clinical course of illness in individual patients [9-11]. The dilemma of differentiating between this three diagnosis, using history, physical examination, radiographic findings and markers of inflammation, is challenging [9-11].

In this study, we want to see the possibility of using standard examinations that are available in daily practice to prevent possible complications and to understand the relationship of parapneumonic effusion with pneumonia.

## Material and Methods

Of 1059 patients with CAP, 304 patients were excluded from the study according to the criteria for exclusion, and because it had realised ultrasound of the pleura and lung as a more sensitive method for the diagnosis of parapneumonic effusion of lung X-ray. Excluding criteria were: cancer and malignant effusion, hospital- acquired pneumonia, solid organ transplanting, transudate effusion, vasculitis, pulmonary emboli (PE), pulmonary tuberculosis and age less than 18 years. From 755 patients, 175 (23.18%), had parapneumonic effusion. Thoracentesis was performed in 94 (53.71%) patients, 50 patients were with uncomplicated parapneumonic effusions (UCPPE) and 44 with complicated parapneumonic effusions (CPPE). Of 580 patients with CAP without effusion, we analysed 148 patients with CAP without effusion because other patients did not have all the necessary laboratory and radiographic parameters to be analysed in this study. The patients were diagnosed and treated in University Infectious Diseases Clinic, Faculty of Medicine, Skopje in the Department of Respiratory Diseases in the period from September 2011 to June 2015.

The demographic characteristics, physical examination findings, laboratory and microbiological findings of all study participants were monitored regularly on University Infectious Diseases Clinic. Initial lung X-rays were taken for all patients at the Institute of Radiology, Medical Faculty in Skopje. After admission, all the patients underwent an ultrasound of the pleura and lung with a three- dimensional echo at the University Infectious Diseases Clinic for diagnosis of pleural effusions and implementation of diagnostic thoracocentesis if the size of effusion was more than 10 mm. After verification of pneumonia and pleural effusion, the distinction between transudation and exudates was done according to Light’s criteria. Exudative pleural effusion is one that meets at least one of the criteria of Light. The transudate if the effusion that meets all three criteria at the same time: 1) to have intercourse protein p/s below 0.5; 2) intercourse LDH p/s below 0.6, and 3) LDH in the pleural fluid under 282 U/L, which is the lowest limit in our laboratory. Than exudative pleural effusion according to their evolution and on the basis of pH, glucose and LDH value in the pleural fluid are division: - Uncomplicated parapneumonic effusions: pH > 7.2, glucose >60 mg/dl, LDH< 1000UI/ml; - Complicated parapneumonic effusions: pH<7.2, glucose <60 mg/dl, LDH>/= 1000 UI/ml.

ERS was determined in all three groups of patients at admission on a clinic. ERS measures the distance through which erythrocytes fall within 1 hour in a vertical tube of anticoagulated blood, and is measured in millimetres/hour. The blood is drawn into a vertical tube anticoagulated with sodium citrate. Leukocyte count (WBC) was also determined in the clinic laboratory by the white blood cell counter (number per microliter). The amount of protein is determined in the same biochemical laboratory with the standard method. C- reactive protein (CRP) was measured by quantitative methods in biochemistry laboratory of the same clinic with quantitative sandwich enzyme heterogeneous test, Ektahem Clinical Chemistry test, an automated biochemical analyser Vitros 250.

### Statistical analysis

Statistical analysis was conducted using SPSS 17 for Windows. The testing of normality in the distribution of the data was used Kolmogorov-Smirnov and Shapiro-Wilk’s W tests. Categorical traits of displayed by absolute and relative representation with quantitative traits mean SD, median, minimum, maximum, 25-75 percentiles. To compare three groups of subjects in relation to the variables analysed were used Kruskal-Wallis ANOVA and Mann-Whitney U TECH (Z). The level of significance or importance was taken the value of p < 0.05, a significantly higher value of p < 0.01.

## Results

In three groups of patients, majority was male patients (58.11%, 58%, and 61.36% consequently). It was insignificant the difference in the distribution of patients with CAP without effusion, UCPPE and CPPE in terms of their sex (p = 0.9). The average age of patients only with CAP was 54.58 ± 17.5 years, in UCPPE 55.5 ± 16.6 and in a group with CPPE was 51.91 ± 18.4 and there was no statistical difference (p = 0.58) (Table 1).

**Table 1:** Demographic characteristics of three groups of patients

Variable	CAP N = 148	UCPPE N = 50	CPPE N = 44	p value
Sex n (%)				
Male	86 (58.11)	29 (58)	27 (61.36)	^a^p = 0.9
Female	62 (41.89)	21 (42)	17 (38.64)	
Age (years) mean ± SD, min-max				
	54.58 ± 17.5 18-89	55.5 ± 16.6 21-83	51.91 ± 18.4 18-93	^b^p = 0.58

a p (Chi-square test);

b p (Analysis of variance).

Analysis of the results regarding the presence of comorbidity showed a significant difference between the three groups of subjects (p = 0.028). Usually with accompanying diseases were CPPE patients (77%), compared with patients with UCPPE (56%) and CAP without effusion (52.7%).

The chronic heart failure was the most common comorbidity in a group with CAP (28; 18.92%) and UCPPE(7; 14%), alcoholism (12;12.77%) in a group with CPPE. The structure of morbidity among the three groups of respondents is presented in Table 2.

**Table 2:** Comorbidity of the three patient groups

Type of comorbidity n (%)	CAP without effusion N= 78 (52.7%)	UCPPE N=28 (56%)	CPPE N=34 (77%)
COPD	8 (5.41)	2 (4)	2 (4.55)
Chronic heart failure	28 (18.92)	7 (14)	2 (4.55)
Diabetes melitus	13 (8.78)	7 (14)	3 (6.82)
Chronic liver disease	2 (1.35)	1 (2)	1 (2.27)
Renal failure	2 (1.35)	1 (2)	0
Alcoholism	4 (2.7)	1 (2)	11 (25)
Malignancy	3 (2.03)	4 (8)	2 (4.55)
Chronic systemic disease	3 (2.03)	1 (2)	2 (4.55)
Poor dental hygiene	0	0	2 (4.55)
Drug users	0	0	2 (4.55)
Neurological disease	3 (2.03)	0	2 (4.55)
Haematological disease	2 (1.35)	0	0
Two or three comorbidities	35 (23.65)	3 (6)	5 (11.36)
Other diseases	7 (4.73)	1 (2)	0

The three groups of participants significantly differ in terms of frequency of occurrence of dyspnea (p = 0.000009). Very high percentage of patients with CPPE had dyspnea (88.64 %), despite the significantly lower percentage of patients with CAP without effusion (47.97%), and significantly lower percent patients with UCPPE (52%). Pleuritic chest pain (86.36%) was the most common symptom and significantly more often in a group with CPPE, then to group with UCPPE (60%), and group with CAP without effusion (25.68%) (Table 3).

**Table 3:** Clinical characteristics of the three groups of patients

Variable	CAP without effusion N = 148 N (%)	UCPPE N = 50 N (%)	CPPE N = 44 N (%)	p-value
	1	2	3	
Fever	107 (72.3)	30 (60)	29 (65.91)	^a^p = 0.2
Catarrhal symptoms	51 (34.46)	16 (32)	11 (25)	^a^p = 0.5
Sore throat	61 (41.22)	15 (30)	10 (22.73)	^a^p = 0.05
Hyperemia of the tonsils and pharynx	78 (52.7)	21 (42)	19 (43.18)	^a^p = 0.3
Cough type				
Productive	43 (29.05)	12 (24)	20 (45.45)	^a^p = 0.049*
Serous sputum	29 (19.59)	15 (30)	3 (6.82)	2vs3 p = 0.005**
Haemoptisis	14 (9.46)	2 (4)	6 (13.64)	
Dry cough	40 (27.03)	15 (30)	7 (15.91)	
Without cough	22 (14.86)	6 (12)	8 (18.18)	
Headache	95 (64.19)	33 (66)	23 (52.27)	^a^p = 0.3
Malaise	138 (93.24)	45 (90)	44 (100)	2vs3 ^a^p = 0.038*
Myalgia	81 (54.73)	23 (46)	24 (54.55)	^a^p = 0.5
Arthralgia	66 (44.59)	19 (38)	17 (38.64)	^a^p = 0.6
Weight loss	76 (51.35)	30 (60)	38 (86.36)	^a^p = 0.0002** 1vs3 p = 0.00003** 2vs3 p = 0.004**
Dispnea	71 (47.97)	26 (52)	39 (88.64)	^a^p = 0.000009** 1vs3 p = 0.00002** 2vs3 p = 0.0001**
Days of disease before hospitalisation				
1 – 4 days	62 (41.89)	23 (46)	9 (20.45)	^a^p < 0.001
5 – 10 days	70 (47.3)	20 (40)	21 (47.73)	1vs3 p = 0.00006
> 10 days	16 (10.81)	7 (14)	14 (31.82)	1vs3 p = 0.001** 2vs3 p = 0.017*
Days of disease before hospitalisation (mean ± SD) median (25-75^th^ quartiles)				
	6.39 ± 6.0	6.24 ± 4.7	10.59 ± 8.8	^c^p = 0.0002**
	5 (3-7)	4 (3-7)	10 (5-13.5)	1vs3 p = 0.00006**
Length of hospitalisation (mean±SD) median (25-75^th^ quartiles)				
	12.39 ± 2.7	15.78 ± 4.3	20.75 ± 18	^c^p < 0.0001
	12 (10-14)	15 (13-16)	21(18-23)	1vs2 p < 0.0001 1vs3 p < 0.0001

a p (Chi-square test);

c p (Kruskal-Wallis test);

* p < 0.05;

** p < 0.01.

Participants from all three groups had a significantly different duration of disease symptoms before hospitalisation (p = 0.0002). This difference was due to a significantly longer duration of symptoms in the group with CPPE compared with the group wit CAP without effusion (p = 0.00006).

The length of hospitalisation significantly differed among the three groups of patients (p < 0.0001). The average time of hospital days shows that half of the patients with CPPE were in the hospital more than 21 days, half of the patients with CAP without effusion were hospitalised more than 12 days, 50% of patients with UCPPE more than 15 days. The results are shown in Table 3.

The values of body temperature before hospitalisation insignificant differed between patients of the three groups (p = 0.07). Again along the duration of fever before hospitalisation was significant among the three groups of subjects (p = 0.0006).

In patients with CPPE average period of the fever duration during hospitalisation is 4 days, in a group with CAP without effusion is 2 days, and in patients with UCPPE is 3 days. Average value of temperature during hospital treatment in patients with CPPE was 39°C (38.25-39.8°C), in patients with UCPPE was 38.4°C (37.5-39.6°C) and the lowest values were noted in the patient with CAP without effusion 38.3°C (37.4-39.2°C) (Table 4).

**Table 4:** Value and duration of temperature in the three patients groups

Variable	CAP without effusion N = 148	UCPPE N = 50	CPPE N = 44	p-value
	1	2	3	
Values of temperatures before hospitalisation, °C [n (%)]				
< 37.1°C	8 (5.41)	0	0	
37.1 – 37.7°C	7 (4.73)	9 (18)	1 (2.27)	
37.8 – 39°C	70 (47.3)	24 (48)	24 (54.55)	
39.1 – 40°C	39 (26.35)	14 (28)	4 (9.09)	
40°C >	24 (16.22)	3 (6)	15 (34.09)	
Values of temperatures before hospitalisation, (mean ± SD) median (25-75^th^ quartiles)				
	38.81 ± 3.1	38.87 ± 0.9	39.37 ± 1.0	^c^p = 0.07
	39 (38.5-40)	39 (38.3-39.8)	39 (38.5-40.5)	
Duration of temperatures before hospitalisation (days) (mean ± SD) median (25-75^th^ quartiles)				
	4.36 ± 4.2	4.0 ± 2.9	7.45 ± 6.4	^c^p = 0.0006**
	4 (2-5.5)	3.5 (3-5)	6 (3-10)	1BC 3 p = 0.0003** 2BC3 p = 0.001*
Duration of temperatures during hospitalisation, °C (days) [n (%)]				
Without fever	92 (62.16)	18 (36)	20 (45.45)	
1 – 2 days	22 (14.86)	18 (36)	6 (13.64)	
3 – 4 days	13 (8.78)	11 (22)	13 (29.55)	
5 – 7 days	9 (6.08)	0	5 (11.36)	
> 7 days	12 (8.11)	3 (6)	0	
Duration of temperatures during hospitalisation (days) mean ± SD median (25-75^th^ quartiles)				
	2.7 ± 3.1	3.3 ± 2.0	4.77 ± 5.0	^c^p = 0.0005**
	2 (1-3)	3 (2-4)	4 (1.5-5)	1BC3 p = 0.003**
Values of temperatures during hospitalisation, °C [n (%)]				
< 37.1°C	23 (15.54)	6 (12)	1 (2.27)	
37.1 – 37.7°C	27 (18.24)	9 (18)	7 (15.91)	
37.8 – 39°C	53 (35.81)	17 (34)	15 (34.09)	
39.1 – 40°C	41 (27.7)	17 (34)	17 (38.64)	
40°C >	4 (2.7)	1 (2)	4 (9.09)	
Values of temperatures during hospitalisation mean ± SD median (25-75^th^ quartiles)				
	38.38 ± 1.1	38.55 ± 1.1	38.9 ± 1.1	^c^p = 0.02*
	38.3 (37.4-39.2)	38.4 (37.5-39.6)	39(38.25-39.8)	1BC3 p = 0.006**

c p (Kruskal-Wallis test);

* p < 0.05;

** p < 0.01.

In the admission to the clinic, patients with CAP without effusion, with UCPPE and CPPE were significantly different ERS rate (p = 0.00090). Patients with CPPE had average ERS in the first hour of 74.77 ± 27.3 mm/h and is significantly higher than the average value in the group with pneumonia without effusion (56.35 ± 28.6), and the average value of the group with UCPPE (60.02 ± 27.7). In the admission, WBC was significantly higher in the group with CPPE versus the group with CAP without effusion (median 12.6 vs. 10.6, p = 0.01). Normal values of WBC had more patients with CAP without effusion (21.62 % vs. 34.09 %), while the values of WBC higher than 20x10^9^/L had more patients with CPPE (25% vs. 10.14%). The values of the inflammatory marker CRP at the admission and discharge on the clinic significantly dependent on the form of manifestation of community-acquired pneumonia (CAP) or its manifestation without effusion, with UCPPE or CPPE (p < 0.0001 and p = 0.04 respectively). The mean values of CRP (231.79 ± 112.2 mg/L) were significantly higher in the group with CPPE compared with the group wit CAP without effusion (139.48 ± 105.7 mg/L) (p = 0.000004), and compared with the group with UCPPE (163.8 ± 147.9 mg/L) (p = 0.000065) (Table 5).

**Table 5:** Markers of inflammation in the three clinical groups of patients

Variable	CAP N = 148	UCPPE N = 50	CPPE N = 44	p value
	1	2	3	
ERS mm/h - admission n (%)				
≤ 20	20 (13.51)	4 (8)	2 (4.55)	
21 – 40	36 (24.32)	8 (16)	6 (13.64)	
41 – 60	37 (25)	19 (38)	8 (18.18)	
61 – 100	52 (35.14)	17 (34)	24 (54.55)	
> 100	3 (2.03)	2 (4)	4 (9.09)	
ERS mm/h mean ± SD median (25-75^th^ quartiles)				
Admission	56.35 ± 28.6	60.02 ± 27.7	74.77 ± 27.3	^b^p = 0.0009**
	60 (33.5-80)	55 (45-80)	76.5 (60-97)	1BC3 p = 0.0004**
Dischagre	34.8 ± 24.0	36.12 ± 22.2	41.73 ± 26.8	2Bc3 p = 0.03*
	31 (11-50)	40 (15-52)	40 (20-60)	^c^p = 0.3
WBC x 10^9^/L n (%)				
≤ 4	13 (8.78)	3 (6)	0	
4 – 9	48 (32.43)	15 (30)	7 (15.91)	
9.1 – 12	32 (21.62)	14 (28)	15 (34.09)	
12.1 – 15	26 (17.57)	3 (6)	6 (13.64)	
15.1 – 20	14 (9.46)	10 (20)	5 (11.36)	
> 20	15 (10.14)	5 (10)	11 (25)	
WBC x 10^9^/L mean ± SD median (25-75^th^ quartiles)				
Admission	11.56 ± 6.8	12.19 ± 6.0	14.68 ± 6.6	^c^p = 0.013*
	10.6 (6.4-14.1)	11.1 (8.1-16.7)	12.6 (10.2-20.2)	1BC3 ^d^p = 0.01*
Dischagre	7.91 ± 2.6	7.26 ± 1.9	7.62 ± 2.8	^b^p = 0.26
	7.6 (6.2-9.3)	7.5 (5.8-8.6)	6.8 (6-8.8)	
CRP serum means ± SD median (25-75^th^ quartiles)				
Admission	139.48 ± 105.7	163.8 ± 147.9	231.79± 112.2	^c^p < 0.0001
	120 (48-228)	120.5 (63-204)	218 (162.5-313)	1BC3 ^d^p = 0.000004**
Dischagre	13.82 ± 22.9	12.6 ± 16.1	32.43 ± 47.3	2BC3 ^d^p = 0.00028**
	6 (3-15)	7 (3-14)	17 (4.5-33.5)	^c^p = 0.04* 1BC3 ^d^p = 0.014*

b p (Analisys of variance);

c p (Kruskal-Wallis test);

d p (Mann-Whitney test);

* p < 0.05;

** p < 0.01.

Radiographic findings regarding the type of infiltration were significantly different between patients with CAP without effusion and CPPE (p = 0.00019), and between the two patient groups with effusion (p = 0.035). Alveolar infiltrates often had as radiographic finding patients with CPPE (65.91 %). Interstitial infiltration was the most common radiographic finding in the group with CAP without effusion (14.86 %), this finding was no patient with CPPE, and the mixed finding was most common in the group with CAP without effusion (51.35%).

Regarding the other two radiographic parameters analysed, distribution by extensiveness and prevalence of pulmonary changes, comparative analysis of the results confirmed the only significant difference between the group with CPPE and the group with CAP without effusion (p = 0.0001, p = 0.00005). Diffuse pulmonary changes were detected more frequently in the group with CAP without effusion compared with the group with CPPE (64.86% vs. 27.27%), while the segment distribution was more common finding in the group with CPPE (50 % vs. stands at 20.27%).

Unilateral localization of the inflammatory process was common finding in the group with CPPE compared with the group with CAP without effusion (79.55% vs. 44.59%), while patients with CAP without effusion were more likely than those with CPPE had radiographic finding of both lungs (55.41% vs. 20:45%) (Table 6).

**Table 6:** Radiographic characteristics of the three groups of patients

Variable	CAP without effusion N = 148	UCPPE N = 50	CPPE N = 44	p-value
	1	2	3	
Type of pulmonary infiltrates n (%)				
Alveolar infiltrates	50 (33.78)	22 (44)	29 (65.91)	^a^p = 0.0012** 1vs3 p = 0.00019**
Interstitial infiltrate	22 (14.86)	4 (8)	0	2vs3 p = 0.035*
Mixed (alveo- interstitial infiltrate)	76 (51.35)	24 (48)	15 (34.09)	
Distribution according to infiltrate extensiveness n (%)				
Diffuse	96 (64.86)	25 (50)	12 (27.27)	^a^p = 0.0007**
Multilocular	3 (2.03)	1 (2)	2 (4.55)	1vs3 p = 0.0001**
Lobar	19 (12.84)	11 (22)	8 (18.18)	
Segmental	30 (20.27)	13 (26)	22 (50)	
Distribution of changes in lung n (%)				
Unilaterally	66 (44.59)	32 (64)	35 (79.55)	^a^p = 0.00008**
Bilaterally	82 (55.41)	18 (36)	9 (20.45)	1vs3 p = 0.00005**

a p (Chi-square test);

c p (Kruskal-Wallis test);

d p (Mann-Whitney test);

* p < 0.05;

** p < 0.01.

## Discussion

Pneumonia can be complicated by the development of parapneumonic effusion, which has increased morbidity and mortality. Aside from inflammation in the lung and pleural space from direct invasion of bacteria and bacteriologic virulence features contributing to parapneumonic effusion, patient’s factors and comorbidities also contribute to the pathophysiology of parapneumonic effusion development.

A recent study [11] analysed 4715 patients with CAP and 882 (19%) had pleural effusion, of which 261 (30%) had complicated parapneumonic effusion or empyema. In this study a multivariable analysis, no single baseline patient’s characteristics distinguished patients without pleural effusion from those with uncomplicated parapneumonic effusion. However, five independent baseline characteristics could predict the development of complicated parapneumonic effusion or empyema: age < 60 years old, alcoholism, pleural pain, tachycardia and leucocytosis. These investigators and others have found a reduced prevalence of clinical manifestation in older patients, suggesting a possible age-related change in the immune response [11-13]. In our study patients with complicated parapneumonic effusion are younger compared with patients with CAP without effusion and those with UCPPE but there is no significant statistical difference regardless of age.

But when it comes to comorbidities, patients with complicated parapneumonic effusion have more comorbidities of patients with CAP without effusion and patients with UCPPE. As in the previously mentioned Falguera’s study, and in Chalmer’s study realised in 2011 [10], in our study also alcoholism was the most common comorbidities which were noted in patients with CPPE, then followed diabetes mellitus. In patients with CAP without effusion and patients with UCPPE most common accompanied disease in this research is chronic heart failure [4, 14]. Having more than one comorbidity proved significant in our study in the development of CPPE [14].

In an earlier study as the most common comorbidity when it comes to CPPE were reported diabetes mellitus, malignancy, and then alcoholism, cardiovascular disease, liver cirrhosis and immunosuppressive states (HIV infection) [5, 12, 15, 16]. In Chapman’s study from 2004 [7] and Finish study from 2014 [3], it is generally accepted that diabetes mellitus increases susceptibility to infection and diabetes is typically included in the list for pleural infection and empyema. Perhaps the explanation for alcoholism, like as a significant risk factor is the existence of anaerobic infections in these patients, is associated with poor dental hygiene and aspiration. CPPE and empyema occur commonly, however, in the absence of any identifiable risk factors [7].

In daily practice, medical doctors first encounter with the clinical features of patients. Unfortunately, the symptoms of pneumonia involving parapneumonic effusion or empyema (i.e fever, malaise, cough, dyspnea and pleural chest pain) are similar to those of pneumonia without a parapneumonic effusion [1, 2, 7]. The symptoms with a parapneumonic effusion can be either acute or chronic [1, 7, 17]. Anaerobic pulmonary infections frequently have an associated pleural effusion and are characterised by a more chronic course [1, 2, 11, 17]. Weight loss and anaemia are common with anaerobic infection [1, 7]. Similarly, if the patients have a parapneumonic effusion, the clinical picture is similar whether or not the effusion is complicated [1].

Very high percentage of patients with CPPE had dyspnea (88.64%), despite the significantly lower percentage of patients with CAP without effusion (47.97%), and significantly lower percent patients with UCPPE (52%). Pleuritic chest pain (86.36%) was the most common symptom and significantly more often in a group with CPPE, then to group with UCPPE (60%), and group with CAP without effusion (25.68%). Weight loss is statistically significantly more frequent in patients with CPPE (86.36%) than in patients with UCPPE (60%), and patients with pneumonia without effusion (51.35%). These results correlate with findings from two major studies of Chalmers and Falguera that pleural chest pain correlated with other factors (clinical, laboratory and microbiological) can predict the development of complicated parapneumonic effusion or empyema [10, 11]. But, the absence of pleural chest pain does not exclude pleural infection [18].

Our results show that significantly different duration of disease symptoms before hospitalisation (p = 0.0002). This difference was due to a significantly longer duration of symptoms in the group with CPPE compared with the group with CAP without effusion (p = 0.00006). The length of illness > 5 days is more common in patients with CPPE, and we found the average length of illness of 10 days (5-13.5 days) in our patients with CPPE [11]. The length of hospitalisation significantly differed among the three groups of patients (p < 0.0001). The average time of hospital days shows that half of the patients with CPPE were in the hospital more than 21 days, half of the patients with CAP without effusion were hospitalised more than 12 days, 50 % of patients with UCPPE more than 15 days. Significantly different length of hospitalisation was reported in several studies [1, 2, 7, 10, 11, 17, 18]. This is due to shortcomings in the management of patients with pneumonia and UCPPE. Early antibiotic treatment will prevent the development of UCPPE and progression to CPPE and empyema. In that case, effusion is associated with antimicrobial treatment failure [1-3], prolonged systematic sepsis, increased the length of hospital stay [2], higher financial costs [2] greater morbidity from invasive procedures [17, 18] and greater mortality [1-3, 7, 10, 11].

We found and that duration of elevated temperature before and after hospitalisation in patients with CPPE significantly different from the length and duration of the temperature in the remaining two groups of patients. Average value of temperature during hospital treatment in patients with CPPE was 39°C (38.25-39.8°C) in patients with UCPPE was 38.4°C (37.5-39.6°C) and the lowest values were noted in the patient with CAP without effusion 38.3°C (37.4-39.2°C). This suggests that the temperature as a clinical marker of inflammation is higher and longer lasting in patients with complicated effusion. A temperature that persists in patients with pneumonia suggests that the inflammatory process takes the adverse course and complicating [19-21].

We decided to see if the standard markers of inflammation, like ERS and WBC, which are widely used in everyday practice, may indicate what course shall take and whether pneumonia complicated by the development of effusion. WBC has an important role in the inflammatory response to infection and the release of pro- inflammatory cytokines [21]. In our study, there were significantly higher values of WBC in patients with effusion especially in patients with CPPE like in study of Kruger and associates [21]. We found that higher value of WBC than 20 x 10^9^ at admission at the clinic are meet in 25% of patients with CPPE because the patients with CPPE have a more severe clinical picture unlike patients with CAP without effusion and patients with UCPPE [1, 8].

Patients with CPPE had average ERS in the first hour of 74.77 ± 27.3 mm/h and is significantly higher than the average value in the group with pneumonia without effusion (56.35 ± 28.6 mm/h), and the average value of the group with UCPPE (60.02 ± 27.7 mm/h). Our result is the same as in growing number of studies, but just like WBC and ESR is a nonspecific marker that indicates not only the severity of the infection but increases in other types of inflammation [22, 23]. ERS is helpful in monitoring chronic inflammatory condition but does not benefit at monitoring responses to therapy in acute inflammatory conditions, such as acute infections [22].

Unlike the previous two markers CRP is more beneficial in acute infections Our results show that the mean values of CRP (231.79 ± 112.2 mg/L) were significantly higher in the group with CPPE compared with the group with UCPPE (163.8 ± 147.9 mg/L) (p = 0.000065), and especially compared with the group with CAP without effusion (139.48 ± 105.7 mg/L) (p = 0.000004). Chalmers et al have reported an association of a low CRP level of >= 100 mg/l at the time of hospital admission with a reduced risk of 30- day mortality, need for mechanical ventilation and/or inotropic support, and complicated pneumonia [24]. Similar results are and Mira in their study published 2008 in a group of patients hospitalised in intensive care unit [25].

Eisenhut’s study from 2008 suggests that a persistently elevated or rising CRP level in a patients on the ICU should, therefore, alert the clinician not only to a potentially poor prognosis but also prompt a reassessment of the patients with a chest X- ray and chest ultrasound for the presence of an empyema that may require surgical evacuation [26]. Alveolar infiltrates with segment distribution and unilateral distribution of changes in the lung are associated with complicated parapneumonic effusion in the conducted study. Unlike them in patients with pneumonia usually meet mixed (also- interstitial infiltrate), according to infiltrate extensiveness diffuse distribution, and bilaterally distribution of changes in the lung.

If you know that about 200 ml of pleural fluid is detectable on PA chest radiography whereas only 50 ml fluid is detectable on a lateral film [8]. Chest radiography with lateral decubitus film in everyday practice is not realised. Very often there is a pleural effusion that classical chest radiography not visualises.

Ultrasound is more accurate and more sensitive for estimable pleural fluid volume and aids thoracocentesis [8, 27, 28]. Ultrasound is also useful in showing separation and echogenicity (correlating with an exudate) and differentiates between the pleural fluid and thickening [8, 27, 28]. It is portable and position flexible [8]. So when we see segmental or lobar pneumonia without recording the effusion is necessary to consider and such a complication and make lung ultrasound.

We can summarise that if we have a patient aged 60 years with a fever that lasts longer, elevated leukocyte, persisting elevated CRP and radiographic findings of segmental or lobar pneumonia should suspect to a parapneumonic effusion as a possible complication, especially CPPE.

In conclusion, perhaps our study will help to clarify some contradictions that are associated with pneumonia and development of parapneumonic effusions, especially CPPE. But, proper analysis of clinical, laboratory and radiographic features, together, of patients with CAP, UCPPE and CPPE to prevent mismanagement of these patients, will reduce morbidity and mortality and help to define new diagnostic and therapeutic approaches.
